# Current Appraisal and Gaps in Knowledge in Cardio–Kidney Metabolic Syndrome Definition

**DOI:** 10.3390/ijms27041657

**Published:** 2026-02-08

**Authors:** Alberto Palazzuoli, Anna Vittoria Mattioli, Francesco Fedele

**Affiliations:** 1 Cardiovascular Diseases Unit, Cardio Thoracic and Vascular Department, Le Scotte Hospital University of Siena, 53100 Siena, Italy; 2 Istituto Nazionale per le Ricerche Cardiovascolari, 40126 Bologna, Italy; 3 Department of Quality of Life Sciences, University of Bologna-Alma Mater Studiorum, 47921 Bologna, Italy; 4 Department of Clinical, Internal, Anesthesiology and Cardiovascular Sciences, Sapienza University of Rome, 00161 Rome, Italy

**Keywords:** cardiovascular diseases, risk factors, cardio–renal syndrome, biomarkers, management

## Abstract

Although metabolic, renal, and cardiovascular disorders frequently coexist, little is known about how illness combinations affect prognosis. Cardiovascular disease (CVD), which can manifest as coronary artery disease (CAD), stroke, heart failure (HF), arrhythmias, and sudden cardiac death, is more likely to develop in patients with chronic kidney disease (CKD). This link is closer with regard of heart failure (HF) and renal dysfunction, in which a reciprocal relationship has been demonstrated, with the initial illness of one organ causing the progressive dysfunction of the other system. Common risk factors for both illnesses include obesity, diabetes, metabolic disorders, hypertension, and dyslipemia. Theoretically, each of these factors accelerates the atherosclerotic process or directly damages the endothelium through inflammatory, oxidative, and pro-thrombotic pathways, which in turn causes the beginning of heart dysfunction and renal function deterioration. Although the mechanisms and causes have been identified, there are still a number of unanswered questions regarding classification, development, monitoring, and preventive aspects. Furthermore, the absence of reliable data on cardiac and renal outcomes across different stages contributes to creating confusion in CKM classification and management. This paper discusses the current challenges and perspectives in CKM definition and assessment proposing a specific diagnostic and laboratory fingerprint.

## 1. Introduction

Traditional cardiovascular risk factors are the initial triggers not only of atherosclerotic disease but also of heart and kidney disease. They also cause a progressive deterioration of cardiovascular and renal function [1]. Chronic kidney disease (CKD) and heart failure (HF) are two conditions with high morbidity and mortality rates and clinical courses that are often silent in the initial stages but may only manifest in the more advanced stages of the disease [2,3]. It is also known that both conditions are associated with a higher cardiovascular risk, and, when they occur simultaneously, the risk of adverse events is further increased. The available epidemiological data only highlight a liaison between risk factors that predispose individuals to the onset and deterioration of both kidney disease and heart failure [4,5]. However, to date, no large-scale studies have explored and compared heart failure, kidney failure, and cardiorenal failure [6,7] (Table 1). Indeed, the combined presence of these factors always results in criteria exclusion in controlled clinical trials. The failure to recognize a specific cardiorenal disease has therefore led to an ineffective prevention and treatment strategy, with a growing need to develop a precise definition of cardiorenal syndrome and its evolution, and to identify specific treatment models focused on harmonizing specific guidelines and current scientific evidence [8].

## 2. A New Strategy for CKM Framing

As recently reported in the American Heart Association’s Presidential Statement, cardiovascular–renal–metabolic syndrome (CKM) is defined as a cluster of conditions attributable to interactions between cardiovascular risk factors such as obesity, diabetes, hypertension, dyslipidemia, and hyperuricemia, which can promote both CKD and cardiovascular disease (CVD), including heart failure, atrial fibrillation, coronary artery disease, stroke, and systemic atherosclerosis [9]. This association has a profound impact on CVD and renal morbidity and mortality, with significant socioeconomic implications, effects on quality of life, and risks for patients affected by these conditions. The high prevalence of cardiorenal and metabolic disorders in the population represents a public health emergency, but it may also provide an opportunity for targeted preventive treatment to reduce burden and a therapeutic target for patients with heart and kidney failure [10]. In this sense, there are currently several therapeutic options that improve the metabolic risk profile and renal function and have cardioprotective effects. In order to improve cardiovascular, renal, and metabolic health, it appears necessary to investigate the following factors: (1) clarify the definition of cardiovascular, renal, and metabolic syndrome; (2) establish a cardiovascular, renal, and metabolic staging strategy that promotes lifelong prevention; (3) promote prevention algorithms that include and define the CVD risk clusters most closely related to cardiorenal events; and (4) identify strategies for the treatment and management of cardiovascular and renal diseases caused by pre-existing metabolic disorders [11,12]. This approach is based on the integration and understanding of the main guidelines through the establishment of longitudinal evidence-based treatment models focused on reducing the fragmentation of various specialties and facilitating interdisciplinary care [12,13]. In this sense, the establishment of a research group could promote the training of physicians who can guarantee specific skills and quality of care for these patients. A Special Issue would provide guidance for the definition, staging, risk profiles, diagnostic algorithms, and therapeutic approaches for these patients, and would also organize a network of expertise involving cardiologists who can broaden their knowledge and interests in diabetes, nephrology, and endocrinology. The aim is to establish a multidisciplinary vision of the syndrome, aiming to improve health and reduce cardiovascular and renal events through a more defined approach. In this regard, a collaboration with other study groups (heart failure, hypertension and prevention, gender-related diseases) could be useful and desirable. Improving diagnostic recognition and follow-up across different stages of CKD requires a stage-specific, multidisciplinary approach that enhances communication, monitoring, and patient engagement [14,15]. For all these reasons, the identification of subtypes and related risk assessment requires a specific laboratory pattern and iconographic tools reflecting the underlying pathophysiological pathways of the diseases (Table 2).

## 3. Traditional and Future Laboratory Panel

Diagnosis and follow-up rely on integrating labs across metabolic, renal, and cardiac domains: metabolic domains account for fasting glucose and glycosylated hemoglobin (HbA1c) insulin levels, Lipid Panels (LDL, HDL, Triglycerides, Non-HDL-C, ApoB optional), high-sensitivity c-reactive protein (hs-CRP), and interleukin 6 (IL-6) as inflammatory biomarkers. Traditional renal biomarker patterns include the assessment of serum creatinine and the glomerular filtration rate (eGFR), Cystatin C, the urine albumin-to-creatinine ratio (ACR), electrolytes (sodium, potassium, Chloride, HCO_3_^−^), and mineral bone metabolism [12,16,17]. Tumor necrosis factor receptors (TNFR1, TNFR2) are strong predictors of diabetic kidney disease progression: uPAR reflects immune activation and predicts CKD and CV events; FGF-23 is involved in phosphate regulation, vascular calcification, plaque growth, and destabilization; Neutrophil gelatinase-associated lipocalin (NGAL), kidney injury molecule 1 (KIM-1), and liver fatty acid-binding protein (L-FABP) are early markers of tubular damage. Finally, the cardiac laboratory fingerprint comprises NT-proBNP or BNP for volume status and HF stage monitoring, High Sensivity Troponin (hs-Tn) as index of cardiac damage, and the soluble suppression of tumorgenicity 2 protein (ST2) as a maker of cardiac inflammation and collagen deposition. Growth Differentiation Factor-15 (GDF-15), an increasingly important cardio–renal–metabolic biomarker, is upregulated during elevated immune response hypossiemic status, oxidative stress, and myocardial injury [18]. By integrating traditional biomarker profiles with precision medicine research it will be possible to recognize more specific mechanisms and metabolic pathways responsible for disease progression. This strategy would permit to capture early, dynamic changes beyond the common used laboratory parameters. Metabolomic profiles investigate small molecules (lipids, amino acids, sugars, organic acids) sampled in both blood and urine samples that reflect real-time metabolic activity. The main pathways include the following: Lipid metabolism: This is evaluated using adipokines and fatty acid modulators. Energy metabolism: Altered glucose, lactate, branched-chain amino acids (BCAAs), and ketone bodies reveal dysregulated glycolysis, insulin resistance, and mitochondrial stress linked to insulin resistance and heart failure [19,20]. Inflammatory metabolites: An altered tryptophan–kynurenine pathway and bile acid metabolism reflect immune and gut microbiome contributions. Uremic toxins screened using Indoxyl sulfate, p-cresyl sulfate, and trimethylamine-N-oxide (TMAO) are linked to kidney dysfunction and cardiovascular events. The integration with proteomic maps investigates the pathway-level dysregulation of fibrosis through the measurement of tissue grow factor (TGF-β), collagen fragments, myeloperrosidases (MMPs), and galectin-3. Markers of endothelial dysfunction: Changes in angiopoietin, VEGF, and adhesion molecules reflect vascular injury. Markers of inflammation and immunity: Complement proteins, C-reactive protein, and cytokine networks show chronic low-grade inflammation. All together, these provide a pathway-level fingerprint of CKM, crucial for developing precision medicine approaches [21,22]. More specifically, studies have identified amino acid modifications involving leucine, isoleucine, and valine in individuals with metabolic syndrome. Additionally, alterations in propionylcarnitine, phenylalanine, and docosapentaenoic acid have been observed in individuals with MetS. Proteomic analysis demonstrated the dysregulation of proteins such as the lysosomal cobalamin transporter ABCD4, vitronectin, and profilin-1. Current metabolite dysfunctions are associated with an altered inflammatory cascade involving tumor necrosis factor alpha (TNFα), interleukin-6 (IL-6), Alpha 2 macroglobulin haptoglobin, alpha-1-antitrypsin, and apolipoprotein A-I [23,24,25]. The better knowledge of detailed metabolomic pathways associated with specific clinical conditions may facilitate diagnostic processes and help in the identification of specific phenotypes.

Detailed laboratory metabolomic and proteomic analyses provide a multi-dimensional profile of CKM biology, revealing functional biochemical changes and structural dysfunction. The combination of precision medicine and big data analysis may also be applied to personalize care, moving away from “one-size-fits-all” approach. This new paradigm may optimize risk stratification, disease monitoring, and target management [23,26,27].

## 4. Differences in the Course of Kidney and Heart Diseases

**Figure 1:**
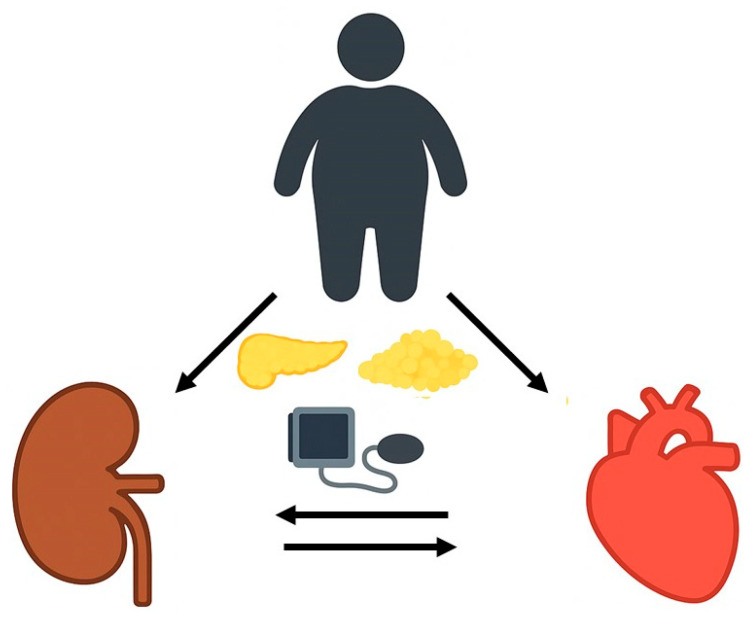
Traditional obese metabolic syndrome with associated CV risk factors leading to a progressive cardiac and renal damage which may reciprocally impair both functions.

The development and interaction of cardiovascular and renal disorders within the cardiorenal–metabolic (CKM) syndrome paradigm exhibits a variety of patterns, processes, and clinical results. The traditional AHA 2023 CKM classification and staging scheme (Stages 0–4) represents an increasing risk and organ involvement across the whole spectrum of cardiac, kidney, and metabolic (CKM) endpoints as an integrated model describing how metabolic risk factors (such as obesity, insulin resistance, and diabetes) promote the interrelated dysfunction of the heart (C) and kidneys (K) [9,12]. Despite the unimodal approach, several issues regarding metabolic triggers, disease sequences, pathophysiological mechanisms, and treatment responses may be elucidated. Notably, some patients progress from more advanced stages of diabetes or dyslipidemia, while others progress from obesity, hypertension, or renal dysfunction. Similarly, in some subjects, kidney dysfunction precedes cardiac remodeling: diabetic nephropathy leads to albuminuria and an increased risk of heart failure. In others, cardiac failure due to hypertensive disease, dilated cardiomyopathy, or coronary arterial disease (CAD) is the initial manifestation, and venous congestion can cause renal damage [16,28]. Even though the current statement confirms their bidirectional nature, cardiac and kidney damage may occur at different rates and times (Figure 1). For example, patients with accelerated atherosclerosis may have preserved cardiac function with a significant impairment in renal function, even in the absence of an acute coronary event; similarly, obese patients with decompensated diabetes may have stiffer myocardial walls and an impaired myocardial relaxation due to increased pericardial fat and intramyocardial collagen deposition, which cause diastolic dysfunction and elevated left ventricular (LV) filling pressure, even though kidney function may remain within normal limits for a long time [29,30]. In this framework, patients with CKM may experience heart failure with a preserved (HFpEF) or reduced ejection fraction (HFrEF) according to the initial cardiac insult, cardiac dysfunction mechanisms, and response to the treatment. Similarly, CKD can occur more frequently because of diabetic nephropathy or glomerulosclerosis related to hypertension, but immunological, inflammatory, or genetic disturbances should also be accounted. Additionally, different risk profiles lead to various degrees of endothelial dysfunction, neurohormonal activation, oxidative stress, and vascular and myocardial fibrosis with related glomerular or tubular damage. The current domains should be accounted for treatment tailoring, along with the variable responses to the administration of different drugs such as Sodium glucose transporter 2 inhibitors (SGLT2i), Glucagon-like peptide-1 (GLP-1) receptor agonists (GLP-1 RAs), Renin angiotensin system blockers (RAASi), and mineralcorticoid receptor antagonists (MRAs) (Table 3). Aside from these differences, other concerns need to be addressed: The first question pertains to the strong relationship between obesity/metabolic syndrome and future cardio–renal problems. Indeed, the prevalence of obesity disease is likely to differ in Europe, Asia, and Africa, as the percentage of people with obesity in these regions differs significantly from that of the United States. It may be worthwhile to refer to patients with a high CV risk burden, with obesity considered an additional disorder included in the CV cluster [31,32]. Accordingly, fat and muscle mass components, as well as waist circumference thresholds, should be measured, and patient’s race, lifestyle, dietary habit and socio-economic condition should be still considered. The primary drawback of the body mass index (BMI) is its failure to reflect visceral adiposity, which is an important predictor of CKM risk. Visceral fat, especially in the abdomen, liver, and heart, is significantly linked to insulin resistance, chronic inflammation, and atherosclerosis. In contrast, subcutaneous fat, especially in the lower body, may not provide the same risk. Individuals with a normal BMI but high visceral fat (so-called “normal-weight obesity”) are more likely to develop metabolic syndrome and have cardiovascular events than those with a greater BMI but less visceral fat. For instance, in Europe, the average BMI distribution implies that many people at risk for CKM are overweight rather than obese [33]. Furthermore, obesity prevalence varies greatly across Europe, with Southern and Eastern countries often having greater rates than Northern or Mediterranean areas [34,35]. This regional diversity, combined with food habits and nutritional patterns such as the Mediterranean diet, may result in a lower overall obesity burden with respect to the United States, but there is a hidden cost of metabolic risk that BMI alone may not reflect [31,36]. Finally, the CKM advisory being strictly focused on CV and metabolic risk does not take into account the presence of primitive cardiomyopathies and valve heart diseases, which are not directly linked to an increased CV risk burden [37,38]. These primitive cardiac disorders can result in cardiac dysfunction and subsequent renal damage due to altered global kidney perfusion, intrarenal blood flow redistribution, neurohormonal activation, and altered hemodynamic conditions [34,35]. According to the conventional HFpEF pattern, CKM appears to refer to individuals who have LV hypertrophy, increased myocardial fibrosis, and vascular stiffness associated with systemic vascular injury [39]. Similarly to this, individuals with CKM usually have intact systolic function along with an increased LV mass, decreased intraventricular elastance, and peripheral calcification.

## 5. Future Research Objectives

The aims of this issue are as follows: 1—The recognition of a homogeneous CKM definition based on epidemiological characteristics and risk profiles through a critical revision of current epidemiological data. 2—The identification and assessment of different subtypes of renal failure that cause cardiovascular complications and heart failure. 3—Understanding the main pathophysiological mechanisms connecting renal and cardiac dysfunctions through a description of current and potential laboratory tools, investigating pathways associated with the main risk factors. 4—To establish an accurate staging of cardiac and renal failure-related progression and the associated social, economic, and health impacts. Indeed, individual evolution should be contemplated for each patient: one subject may initially develop a more severe CKD, with a subsequent HF appearance. Conversely, another subject with initial heart disease may present with HF, and consequently CKD. Thus, the two disorders may not manifest with a simultaneous evolution. 5—The recognition of different heart failure phenotypes related to renal function deterioration, their heterogeneity, and patterns with a poorer prognostic impact. 6—A knowledge of lifestyle, race, dietary habits, and potential triggers across the world related to obesity, diabetes, and other CV risk factors leading to simultaneous cardiac and renal function impairment.

## 6. Conclusions

Due to the current inconsistencies and gaps, future studies can be tailored to the identification of CKM subtypes by addressing the risk stratification using imaging, biomarkers, and multi-omics. The integration between traditional clinical and laboratory markers and metabolomic biomarkers may reveal distinct metabolic substrates corresponding to CKM severity. This approach may also reveal a marked heterogeneity within the CKM cluster. The extensive analysis of large scale populations may enhance risk stratification and favor more personalized management strategies beyond conventional CKM classification. Finally, the application of machine learning or cluster analysis may better define the multifaceted aspects of CKM.

## Figures and Tables

**Table 1:** Main clinical trials investigating patients with high cardiovascular risk or with kidney dysfunction demonstrating reduction in renal events but endpoints and baseline renal characteristics differ each other (T2D type 2 diabetes, CV cardiovascular, CKD chronic kidney disease, LVEF left ventricular ejection fraction, HFpEF heart failure with preserved ejection fraction, HFrEF heart failure with reduced ejection fraction).

**Studies**	**Population Characteristic**	**Total N (Male)**	**Renal Cut-Offs—eGFR (mL/min/1.73 m^2^)**	**Renal Cut-Offs—Albuminuria (UACR, mg/g)**	**Drug Studied**
**FIGARO-DKD**	T2D, CKD (stage 2–4)	7437 (5105)	>25	30–5000	Finerenone
**FIDELIO-DKD**	T2D and advanced CKD	5734 (3841)	>25	30–5000	Finerenone
**FINEARTS-HF**	HF with LVEF ≥ 40%	6001	Not specified (mean ± sd = 62 ± 20)	Not specified	Finerenone
**EMPA-REG OUTCOME**	T2D, high CV risk	7020 (2030)	≥20 a <45 o ≥45 a <90 with albuminuria	≥200	Empagliflozin
**DECLARE–TIMI 58**	T2D, high CV risk	17160 (10639)	≥60	Not specified	Dapagliflozin
**EMPA-KIDNEY**	CKD with/without T2D	6600	20–45	Median ~330	Empagliflozin
**CREDENCE**	CKD and T2D	4401	30–90	Median 927	Canaglifozin
**DAPA-CKD**	CKD with/without T2D	4304 (2880)	25–75	200–5000	Dapagliflozin
**PARAGON-HF**	HFpEF/HFrEF, CKD/diabetes/ASCVD	4800 (2400)	Not specified	Not specified	Sacubitril/ Valsartan
**LEADER**	T2D, high CV risk	9300 (5952)	Not specified	Not specified	Liraglutide
**FLOW**	T2D, CKD	3500	25–75	200–5000	Semaglutide

**Table 2:** Unresolved points and gray zones with related clinical evolution and outcome (CRS cardio renal syndrome, CKM cardio kidney metabolic syndrome, AKI acute kidney injury, HF heart failure, CKD chronic kidney disease).

	**CRS Statement**	**CKM Statement**
**Definition**	Two diseases: renal dysfunction and heart failure	Atherosclerotic manifestations as part of the syndrome
**Classification**	Four patterns: two acute and two chronic	Continuum deterioration
**Cardio-kidney correlation**	Bidirectional nature identified in clinical manifestations of cardio-renal dysfunction	Includes subjects at risk and with sub-clinical evidence of disease
**Temporal assessment**	Mutual chronological relationship between acute HF-AKI and chronic HF-CKD and vice versa	Chronic progressive disease starting from common risk factors from cardiovascular and renal events
**Management aims**	To reduce kidney damage and improve cardiac function	Avoiding advanced cardiac and kidney deterioration

**Table 3:** Current CKM approach and definition and potential researches application to better define disease risk and progression (CKM cardio kidney metabolic syndrome, GFR glomerular filtration rate, CV cardiovascular).

**Current CKM Framework**	**Prospective Research**
Cardiovascular and kidney progression arising from metabolic dysfunctions	Recognize specific CV risk factors and mechanisms responsible for cardiac and kidney damage
Obesity classification based on BMI	Expand the obesity definition including race lifestyle dietary habits and visceral fat assessment
Generic cardiac and kidney dysfunction damage proceeding in simultaneous manner	Identification of initial cardiac and kidney triggers responsible for future dysfunction
Staging classification which reflects disease progression	Address specific diagnostic algorithm including laboratory and imaging tools to better define risk stratification
Cardio renal risk defined by traditional cardiovascular risk factors associated with albuminuria and GFR	Research additive biomarkers and multi-omics assays capturing main biological pathways related to inflammation endothelial dysfunction accellerated atherosclerosis
CKM view by cardio-renal damage progression and stage	Definition of different CKM clusters based on initial CV risk, cardiac and kidney remodeling, and clinical manifestations

## Data Availability

No new data were created or analyzed in this study. Data sharing is not applicable to this article.

## References

[1] McAlister F.A., Ezekowitz J., Tarantini L., Squire I., Komajda M., Bayes-Genis A., Gotsman I., Whalley G., Earle N., Poppe K.K. (2012). Meta-analysis Global Group in Chronic Heart Failure (MAGGIC) Investigators. Renal dysfunction in patients with heart failure with preserved versus reduced ejection fraction: Impact of the new Chronic Kidney Disease-Epidemiology Collaboration Group formula. Circ. Heart Fail..

[2] Damman K., Valente M.A., Voors A.A., O’Connor C.M., van Veldhuisen D.J., Hillege H.L. (2014). Renal impairment, worsening renal function, and outcome in patients with heart failure: An updated meta-analysis. Eur. Heart J..

[3] Mentz R.J., Anker S.D., Pitt B., Rossing P., Ruilope L.M., Gebel M., Kolkhof P., Lawatscheck R., Rohwedder K., Bakris G.L. (2025). Efficacy and safety of finerenone in patients with chronic kidney disease and type 2 diabetes by diuretic use: A FIDELITY analysis. Eur. J. Heart Fail..

[4] McCullough P.A., Amin A., Pantalone K.M., Ronco C. (2022). Cardiorenal Nexus: A Review with Focus on Combined Chronic Heart and Kidney Failure, and Insights from Recent Clinical Trials. J. Am. Heart Assoc..

[5] Ronco C., McCullough P., Anker S.D., Anand I., Aspromonte N., Bagshaw S.M., Bellomo R., Berl T., Bobek I., Cruz D.N. (2010). Cardio-renal syndromes: Report from the consensus conference of the acute dialysis quality initiative. Eur. Heart J..

[6] Manjunath G., Tighiouart H., Ibrahim H., MacLeod B., Salem D.N., Griffith J.L., Coresh J., Levey A.S., Sarnak M.J. (2003). Level of kidney function as a risk factor for atherosclerotic cardiovascular outcomes in the community. J. Am. Coll. Cardiol..

[7] Waijer S.W., Vart P., Cherney D.Z.I., Chertow G.M., Jongs N., Langkilde A.M., Mann J.F.E., Mosenzon O., McMurray J.J.V., Rossing P. (2022). Effect of dapagliflozin on kidney and cardiovascular outcomes by baseline KDIGO risk categories: A post hoc analysis of the DAPA-CKD trial. Diabetologia.

[8] Zannad F., Rossignol P. (2018). Cardiorenal Syndrome Revisited. Circulation.

[9] Ndumele C.E., Rangaswami J., Chow S.L., Neeland I.J., Tuttle K.R., Khan S.S., Coresh J., Mathew R.O., Baker-Smith C.M., Carnethon M.R. (2023). Cardiovascular-Kidney-Metabolic Health: A Presidential Advisory from the American Heart Association. Circulation.

[10] Sebastian S.A., Padda I., Johal G. (2024). Cardiovascular-Kidney-Metabolic (CKM) syndrome: A state-of-the-art review. Curr. Probl. Cardiol..

[11] Shlipak M.G., Massie B.M. (2004). The Clinical Challenge of Cardiorenal Syndrome. Circulation.

[12] Zoccali C., Mallamaci F., Halimi J.M., Rossignol P., Sarafidis P., De Caterina R., Giugliano R., Zannad F. (2024). From Cardiorenal Syndrome to Chronic Cardiovascular and Kidney Disorder: A Conceptual Transition. Clin. J. Am. Soc. Nephrol..

[13] Marassi M., Fadini G.P. (2023). The cardio-renal-metabolic connection: A review of the evidence. Cardiovasc. Diabetol..

[14] Schefold J.C., Filippatos G., Hasenfuss G., Anker S.D., von Haehling S. (2016). Heart failure and kidney dysfunction: Epidemiology, mechanisms and management. Nat. Rev. Nephrol..

[15] Khan S.S., Coresh J., Pencina M.J., Ndumele C.E., Rangaswami J., Chow S.L., Palaniappan L.P., Sperling L.S., Virani S.S., Ho J.E. (2023). Novel Prediction Equations for Absolute Risk Assessment of Total Cardiovascular Disease Incorporating Cardiovascular-Kidney-Metabolic Health: A Scientific Statement from the American Heart Association. Circulation.

[16] Khan S.S., Matsushita K., Sang Y., Ballew S.H., Grams M.E., Surapaneni A., Blaha M.J., Carson A.P., Chang A.R., Ciemins E. (2024). Chronic Kidney Disease Prognosis Consortium and the American Heart Association Cardiovascular-Kidney-Metabolic Science Advisory Group. Development and Validation of the American Heart Association’s PREVENT Equations. Circulation.

[17] Matsushita K., Kaptoge S., Hageman S.H.J., Sang Y., Ballew S.H., Grams M.E., Surapaneni A., Sun L., Arnlov J., Bozic M. (2023). Including measures of chronic kidney disease to improve cardiovascular risk prediction by SCORE2 and SCORE2-OP. Eur. J. Prev. Cardiol..

[18] Kosyakovsky L.B., de Boer R.A., Ho J.E. (2024). Screening for Heart Failure: Biomarkers to Detect Heightened Risk in the General Population. Curr. Heart Fail. Rep..

[19] Cabała S., Herosimczyk A. (2025). Diet-Induced Proteomic and Metabolomic Signatures in Chronic Kidney Disease: A Precision Nutrition Approach. Metabolites.

[20] Zhou M., Sun W., Gao Y., Jiang B., Sun T., Xu R., Zhang X., Wang Q., Xuan Q., Ma S. (2025). Metabolomic profiling reveals interindividual metabolic variability and its association with cardiovascular-kidney-metabolic syndrome risk. Cardiovasc. Diabetol..

[21] Liu Y., Sun M., Sun J., Lin F., Xu D., Chen Y., Song W., Li Q., Jiang Y., Gu J. (2024). Identification of novel serum metabolic signatures to predict chronic kidney disease among Chinese elders using UPLC-Orbitrap-MS. J. Nutr. Health Aging.

[22] Cañadas-Garre M., Anderson K., McGoldrick J., Maxwell A.P., McKnight A.J. (2019). Proteomic and metabolomic approaches in the search for biomarkers in chronic kidney disease. J. Proteom..

[23] da Silva C.V.F., da Silva C.J.F., Cataldi T.R., Labate C.A., Sade Y.B., Scapin S.M.N., Thompson F.L., Thompson C., Silva-Boghossian C.M.D., de Oliveira Santos E. (2025). Proteomic and Metabolomic Interplay in the Regulation of Energy Metabolism During Obesity and Metabolic Syndrome. Diabetes Metab. Res. Rev..

[24] Ferreira da Silva C.V., da Silva C.J.F., Bacila Sade Y., Naressi Scapin S.M., Thompson F.L., Thompson C., da Silva-Boghossian C.M., de Oliveira Santos E. (2024). Prospecting Specific Protein Patterns for High Body Mass Index (BMI), Metabolic Syndrome and Type 2 Diabetes in Saliva and Blood Plasma from a Brazilian Population. Proteom. Clin. Appl..

[25] Panov A., Mayorov V.I., Dikalov S. (2022). Metabolic Syndrome and β-Oxidation of Long-Chain Fatty Acids in the Brain, Heart, and Kidney Mitochondria. Int. J. Mol. Sci..

[26] Howell C.R., Zhang L., Mehta T., Wilkinson L., Carson A.P., Levitan E.B., Cherrington A.L., Yi N., Garvey W.T. (2024). Cardiometabolic Disease Staging and Major Adverse Cardiovascular Event Prediction in 2 Prospective Cohorts. JACC Adv..

[27] Lu Y., Ge J., Zhu L., Wang L., Wu J., Dong F., Deng J. (2025). Cardiometabolic-kidney indices and machine learning model for predicting all-cause mortality in patients with cardiovascular-kidney-metabolic syndrome: A longitudinal cohort study. Am. J. Nephrol..

[28] Rangaswami J., Shlipak M.G., Mathew R.O., Ndumele C.E. (2025). The Cardiovascular-Kidney-Metabolic Health Framework: Implications for Nephrology. Clin. J. Am. Soc. Nephrol..

[29] Diamantidis C.J., Zepel L., Wang V., Smith V.A., Hudson Scholle S., Tamayo L., Maciejewski M.L. (2021). Disparities in Chronic Kidney Disease Progression by Medicare Advantage Enrollees. Am. J. Nephrol..

[30] Lassen M.C.H., Ostrominski J.W., Claggett B.L., Neuen B.L., Beldhuis I.E., Butt J., Biering-Sørensen T., Desai A.S., Lewis E.F., Jhund P.S. (2025). Obesity and Risk of Kidney Outcomes in Heart Failure With Preserved Ejection Fraction: A Participant-Level Pooled Analysis of 4 Contemporary Trials. JACC Heart Fail..

[31] Powell-Wiley T.M., Poirier P., Burke L.E., Després J.P., Gordon-Larsen P., Lavie C.J., Lear S.A., Ndumele C.E., Neeland I.J., Sanders P. (2021). Obesity and cardiovascular disease: A scientific statement from the American Heart Association. Circulation.

[32] Laddu D., Neeland I.J., Carnethon M., Stanford F.C., Mongraw-Chaffin M., Barone Gibbs B., Ndumele C.E., Longenecker C.T., Chung M.L., Rao G. (2024). Implementation of Obesity Science into Clinical Practice: A Scientific Statement From the American Heart Association. Circulation.

[33] Janssen F., Bardoutsos A., Vidra N. (2020). Obesity Prevalence in the Long-Term Future in 18 European Countries and in the USA. Obes. Facts.

[34] Vidra N., Trias-Llimós S., Janssen F. (2019). Impact of obesity on life expectancy among different European countries: Secondary analysis of population-level data over the 1975–2012 period. BMJ Open.

[35] Wexler D.J., Garvey W.T., Ghosh A., Kazemi E.J., Krause-Steinrauf H., Ahmann A.J., Brown-Friday J., Casula S., Cherrington A.L., Elasy T.A. (2025). Weight gain was associated with worsening glycemia and cardiovascular and kidney outcomes in patients with type 2 diabetes independent of diabetes medication in the GRADE randomized controlled trial. Diabetes Care.

[36] Marx-Schütt K., Cherney D.Z., Jankowski J., Matsushita K., Nardone M., Marx N. (2025). Cardiovascular disease in chronic kidney disease. Eur. Heart J..

[37] Shahid I., Philip J., Avenatti E., Laddu D., Shapiro M.D., Khera A., Pandey A., Ndumele C.E., Gulati M., Nasir K. (2025). Lifestyle Interventions in Cardiovascular-Kidney-Metabolic Syndrome JACC: Advances Expert Panel. JACC Adv..

[38] Romeo S., Vidal-Puig A., Husain M., Ahima R., Arca M., Bhatt D.L., Diehl A.M., Fontana L., Foo R., Frühbeck G. (2025). Clinical staging to guide management of metabolic disorders and their sequelae: A European Atherosclerosis Society consensus statement. Eur. Heart J..

[39] Palazzuoli A., Caravita S., Paolillo S., Ghio S., Tocchetti C.G., Ruocco G., Correale M., Ambrosio G., Perrone Filardi P., Senni M. (2021). Current gaps in HFpEF trials: Time to reconsider patients’ selection and to target phenotypes. Prog. Cardiovasc. Dis..

